# Common Variable Immunodeficiency Associated with Inflammatory Bowel Disease and Type I Diabetes

**DOI:** 10.4137/ccrep.s3432

**Published:** 2009-11-27

**Authors:** Branka Filipović, Zorica Šporčić, Tomislav Randjelović, Goran Nikolić

**Affiliations:** 1 Department of Gastroenterohepatology Clinical and Hospital Center “Bezanijska Kosa”, Belgrade, Serbia.; 2 Department of Clinical Alergology and Immunology Clinical and Hospital Center “Bezanijska Kosa”, Belgrade, Serbia.; 3 School of Medicine, Surgery Faculty, Belgrade, Serbia. Email: branka.filipovic3@gmail.com

**Keywords:** immunodeficiency, inflammatory bowel disease, diabetes mellitus type I, perianal fistula

## Abstract

Common variable immunodeficiency (CVID) is a heterogeneous group of primary immunodeficiency disorders characterized by defective antibody production, low levels of serum immunoglobulins and increased susceptibility to infection. The patient was a 39-year-old male who was admitted to the gastroenterology department with a two week history of diarrhea, blunt abdominal pain below the umbilicus, prolonged febrile state, loss of appetite and loss of body weight of 18 kg during the previous six months. Screening tests of serum immunoglobulins showed decreased concentrations of three types of immunoglobulins: IgA < 0.24 g/L, IgM < 0.18 g/L and IgG < 1.55 g/L. Lymphocytes immunophenotypisation revealed inversed CD4^+^/8^+^ T cells ratio, 0.31 and absence of plasma cells (CD138 negative). Colonoscopy showed a rectal mucosa like cobblestones with multiple longitudinal and serpentinous ulceration, without involvement of other segments of the colon and the small intestine. Histopathology revealed aphtous ulcerative lesions, transmural inflammation with multiple lymphoid aggregates and benign lymphoid nodular hyperplasia of the small intestine. Plasma cells were absent from the lamina propria. Magnetic resonance imaging of a perianal fistula demonstrated a trans-sphicteric type. This case is specific because of the three illnesses associated and only one case of an association of diabetes mellitus type I and immunodeficiency reported thus far.

## Introduction

Common variable immunodeficiency (CVID) or acquired hypogammaglobulinemia is a heterogeneous group of primary immunodeficiency disorders characterized by defective antibody production, low levels of serum immunoglobulins and increased susceptibility to infection, although, a number of defects of T-cell function and deficits in the memory B-cell have been identified, but the etiology of these defects remains unknown.^1^,^2^ There is, however, a broad spectrum of clinical manifestations including recurrent infections of the respiratory tract and chronic lung disease, various autoimmune pathology, gastrointestinal disease, granulomatous infiltrative diseases, lymphoproliferative disorders and malignancies.^3^–^6^ About 20% of CVID patients developed different gastrointestinal pathology, such as inflammatory bowel disease, protein-loss enteropathy, spru-like syndrome, chronic enteritis caused by giardia or campylobacter infection, celiac sprue.^7^–^10^ An association of diabetes and primary hypogammaglobulinemia is uncommon and has been documented only sporadically in medical literature.^1^,^11^,^12^ In this present paper the authors discuss a case of common variable immunodeficiency associated with inflammatory bowel disease and well-regulated insulin-dependent diabetes, that has not been reported, to our knowledge, in this part of Europe so far.

## Case Report

The patient was a 39-year-old male who was admitted to the gastroenterology department with a two week history of diarrhea (15 bowel movements per day), blunt abdominal pain below the umbilicus, prolonged febrile state, loss of appetite and body weight of 18 kg during last six months. He faced recurrent respiratory infections, otitis media, and sinusitis starting from adolescence. His medical history included enteroviral meningoencephalitis two years prior, right pleural empyema in 1998, and recurrent bilateral inguinal abscesses. Diagnosis of insulin-dependent diabetes was established in 1990. In his family history there was no evidence of any type of hereditary immunodeficiency or autoimmune disease. Upon admittance, the general physical examination revealed cachexia (BMI: 16 kg/m^2^), remarkable pallor and febrile state (38 °C). On palpation, no enlargement of the liver, spleen or peripheral lymph nodes was evidenced. Routine laboratory parameters were found to be normal, except markers for inflammation (erythrocyte sedimentation rate 100 at the end of first hour, fibrinogen: 8, 0 g/L, C reactive protein: 130 mg/L), hypochromic anemia (hemoglobin: 98 g/L, hematocrit: 30%, mean corpuscular volume (MCV): 79 fL, iron concentration in the blood 6.4 μmol/L, iron binding capacity 80 μmol/L, saturation 8%), and malnourishment (total proteins: 49 g/L, serum albumins: 28 g/L, cholesterol level: 3.8 mmol/L and triglycerides: 0.8 mmol/L). There was no serum paraprotein and no IgG was detected in the urine. Protein excretion on a 24-hour urine collection was 0.026 g. The relevant tumor markers did not exceed accepted normal ranges. Stool examination for occult blood, enteric pathogens, ova and parasites were negative. Microbiological analysis excluded some viral infections (hepatitis B, C and HIV). Repeated hemocultures were negative. A nose swab culture was negative. A sputum culture and throat swab cultured positive for *Streptococcus pneumoniae*. Standard immunological markers (ANA, AMA, AGMA, ANCA, antids DNA, crioglobulins, and rheumatoid factor) were excluded for autoimmune and rheumatoid diseases. Screening tests of serum immunoglobulins showed decreased concentrations of three types of immunoglobulins: IgA < 0.24 g/L, IgM < 0.18 g/L and IgG < 1.55 g/L. Lymphocyte immunophenotypisation revealed inversed CD4^+^/8^+^ T cells ratio, 0.31 and absence of plasma cells (CD138 negative). Subpopulation of CD8 T cells did not express CD28. No evidence of genetic defects was established. Chest and abdominal X-rays were unremarkable. Echocardiography excluded infectious endocarditis. The whole body computerized tomography (CT) scans with contrast, demonstrated bilateral inguinal fibrous tissue as a result of recidivated abscesses. Barium based contrast radiography did not show any abnormalities. Colonoscopy revealed rectal mucosa with a cobblestone like appearance with multiple longitudinal and serpentinous ulceration, with no involvement of other segments of the colon and the small intestine. Histopathology revealed aphtous ulcerative lesions, transmural inflammation with multiple lymphoid aggregates and benign lymphoid nodular hyperplasia of the small intestine. Plasma cells were absent from the lamina propria. Magnetic resonance imaging of perianal fistula demonstrated a trans-sphicteric type. Gastroduodenoscopy showed inflammation of the mucosa of the antrum, without gastric and duodenal ulcer or erosions. The endoscopically taken biopsy of the stomach demonstrated chronic active gastritis. The *Helicobacter pylori* test result was positive.

During the stationary period the patient were treated with metronidazol (1000 mg per day), mesalazine (4.8 g/day) during 6 weeks, combined peroral and parenteral multiple antibiotics, and other symptomatic rehydration therapy. The patient received replacement therapy with intravenous immunoglobulin at three weekly intervals at dosage of 400 mg/kg. One year later, the patient was free of infection and the IgG level was consistently higher than 7.5 g/L.

## Discussion

In this report, the authors present an uncommon case where a patient suffered by three immunological deficit based diseases: common variable immunodeficiency, inflammatory bowel disease and type 1 diabetes. This combination of diagnoses has not previously been reported in this region of Europe. The first paper ever reported on a patient with common variable immunodeficiency was published by Janeway in 1953.^12^ Although the incidence of primary immunodeficiency is low, the prevalence of CVID varied, and it ranged from 1/500 to 1/500 000 in the general population.^2^ In one prospective study which included 240 patients with primary hypogammaglobulinemia in the United Kingdom, Hermaszewski and Webster^11^ reported only two patients with mature onset diabetes and none with type I diabetes. Johnston and Virgo^12^ published the first case of the association between CVID and type 1 diabetes. In our patient, diabetes mellitus masked the origin of the recurrent infections and indicated that the primary hypogammaglobulinemia remained undisclosed for a longer period. The age of onset of the symptoms is variable, but predominantly presenting in individuals of both sexes, between 15 and 40 years of age.^1^,^11^,^14^ In most patients genetic defects causing immunodeficiency are not known and only in about 10% of individuals, mutations in genes which predispose CVID have been identified.^15^–^17^ In our case we did not detect any genetic abnormalities. Patients with acquired hypogammaglobulinemia expressed heterogeneous clinical manifestations. Most of them faced recurrent respiratory infections, due to encapsulated bacteria, especially pneumonia, bronchitis, sinusitis, pharyngitis, and otitis.^18^,^19^ Microorganisms most commonly isolated from sputum cultures were *Streptococcus pneumonia*, *Haemophilus influenza* and *Staphylococcus aureus.*^16^,^20^ Other infections which include septicemia and enteroviral meningoencephalitis are rare in patients on adequate immunoglobulin replacement.^18^,^21^ About 20% of individuals with CVID have chronic gastrointestinal symptomatology including bloating and mild diarrhea, but the minority of patients develop severe enteropathy with malabsorption. 22–25 Giardia and campylobacter enteritis occur more frequently in these patients and tend to be more resistant to treatment.^15^,^25^,^26^ Some authors claimed that chronic inflammatory bowel disease might be the first clinical manifestation of unrecognized primary immunodeficiency.^7^,^9^ Many authors have reported that at least 20% of patients with CVID suffered from chronic active gastritis and also found a high incidence of *Helicobacter pylori* colonization, atrophy with intestinal metaplasia and gastric cancer.^27^–^31^ Pathology of the gastrointestinal tract in patients with CVID showed a wide spectrum of histological patterns which could mimic lymphocytic colitis, collagenous enterocolitis, celiac disease, granulomatous disease, lymphocytic gastritis and inflammatory bowel disease.^32^,^33^ Nodular lymphoid hyperplasia, diffuse lymphoid infiltration and loss of intestinal villi have been revealed as the most frequent histopathological findings. The definitive diagnosis of CVID involves histopathological findings, clinical and immunological correlates.^13^,^26^,^32^–^34^ Lymphocyte immunophenotypisation in our patient demonstrated severe panhypogammagobulinemia with evidence of partially compromised cellular immunity. Haymore et al^34^ suggested that decreased numbers of switched memory B cells correlate with lower serum IgG levels and increased rates of autoimmune disease along with recurrent infections of the respiratory and gastrointestinal tracts. Therapy for CVID is always long term intravenous immunoglobulin replacement in standard doses of 200–400 mg/kg every three or four weeks, which reduces frequencies of infections.^35^,^36^ Some authors claimed that administration of tumour necrosis factor antagonists and anti-CD20 immunomodulators have show some efficacy to prevent autoimmunity and inflammation in the small group of patients with CVID.^37^–^39^

In conclusion, common variable immunodeficiency is a rare primary immunodeficiency disorder which represents a diagnostic challenge for every physician. The presence of diabetes mellitus type 1 and inflammatory bowel disease could mimic the essential disease, CVID, and might mislead the physician for a period and lead to the exclusive treatment of either IBD or DM, neglecting the possibility of the parallel global immunodeficiency existence.
